# Incubation of food craving is independent of macronutrient composition

**DOI:** 10.1038/srep30900

**Published:** 2016-08-03

**Authors:** Rebecca A. Darling, Paige M. Dingess, Kevin C. Schlidt, Erin M. Smith, Travis E. Brown

**Affiliations:** 1 University of Wyoming, Neuroscience Program, Laramie, WY 82071, USA; 2 University of Wyoming, School of Pharmacy, Laramie, WY 82071, USA

## Abstract

Cues previously paired with rewarding stimuli induce a time-dependent increase in the motivational craving state (incubation of craving). Whether there is an increase in craving for high-fat (HF) food over time, which may contribute to overeating and obesity, has not been determined. We hypothesized that cues paired with HF pellets would elicit a greater incubation of craving effect than those paired with standard chow (SC) pellets. Rats exposed to cues associated with either HF or SC pellets demonstrated equivalent levels of craving over an abstinence period of 30 days. Diet preference tests between SC pellets and LabDiet revealed that SC pellets were preferred over LabDiet. Rats reared on SC pellets exclusively, did not display incubation of craving for SC pellets, suggesting that prior history with the food plays an important role in cue-induced seeking behavior. Results identified cues previously associated with food undergo a comparable magnitude of incubation of craving. When ingestive behavior was measured after 30 days of abstinence, rats significantly increased their consumption of HF pellets. Our results indicate that food cues gain importance over time, trigger increased approach behaviors, and increased consumption of HF food following abstinence. This may contribute to overeating and the development of obesity.

Environmental cues, which can manifest in many forms, are vastly important for driving behavior. One of the hallmarks of drug addiction is the physiological and subjective craving that follows re-exposure to drug-associated cues (e.g., sights, sounds, smells, etc.), which can facilitate drug relapse^1^,^2^. Seminal work by Grimm *et al*. (2001) demonstrated that operant responding for cocaine-associated cues increases over time, a finding they termed “incubation of craving”^3^,^4^. Subsequently, numerous studies have shown that several drugs of abuse have the potential to induce an incubation of craving effect, including methamphetamine^5^,^6^,^7^, nicotine^8^, heroin^9^, and alcohol^10^. Furthermore, the incubation of craving paradigm is not specific to drug-associated cues and can be applied to food-related cues as well. For example, both sucrose^11^,^12^,^13^,^14^ and saccharin^15^, a non-caloric sweetener, have been shown to produce an incubation of craving effect. This phenomenon has also been observed for palatable food pellets, even when paired with an aversive food shock^6^. In a quantitative meta-analysis of several human studies, Boswell & Kober (2015) determined that exposure to food-related cues and the subjective experience of craving influences and predicts eating behavior and weight gain^16^. Such cues are even sufficient to promote seeking and consummatory behavior when participants are already satiated^17^ and appear to be most salient in obese populations^18^,^19^. Together these studies suggest that food-related cues may underlie or at least contribute to the development of obesity. However, to our knowledge, no studies have examined the potential for high-fat (HF) paired cues to induce a time-dependent increase in food seeking behaviors (incubation effect).

To test our hypothesis that cues associated with a HF reward would display an incubation of craving effect, we utilized a food self-administration model, in which rats either lever pressed for 60% HF pellets or standard chow (SC) pellets. The SC pellets were chosen for comparison because they resembled the macronutrient profile of the home cage diet (LabDiet). We confirmed that the HF pellets were preferred over the SC pellets with a pellet preference test and a conditioned place preference (CPP) assay.

As hypothesized, cues paired with the HF reward elicited incubation of craving, measured by the increased lever responding from Test 1 to Test 2. To our surprise cues paired with the SC pellets demonstrated an equivalent incubation, an effect that was independent of satiation state. However, further analysis revealed that the SC pellet was actually preferred over the home cage LabDiet, despite comparable macronutrient profiles. To further investigate this finding, we utilized a SC pellet-raised control, in which animals were exclusively reared on SC pellets from weaning onward, rather than the standard LabDiet. These animals did not exhibit an incubation of craving effect for SC pellets, evidenced by the lack of increase in lever responding from Test 1 to Test 2, suggesting that reward value is critical to the development of this phenomenon. Although both SC and HF paired-cues induced equivalent levels of incubation, only HF elicited a time-dependent increase in consumption. Together our results indicate that while food cues gain importance over time, regardless of macronutrient composition, exposure to HF following a period of abstinence is sufficient to increase consumption, which may underlie challenges in dieting and facilitate the development of obesity.

## Results

### Experiment 1: Rats show a time-dependent increase in consumption of high-fat pellets

**Figure 1:**
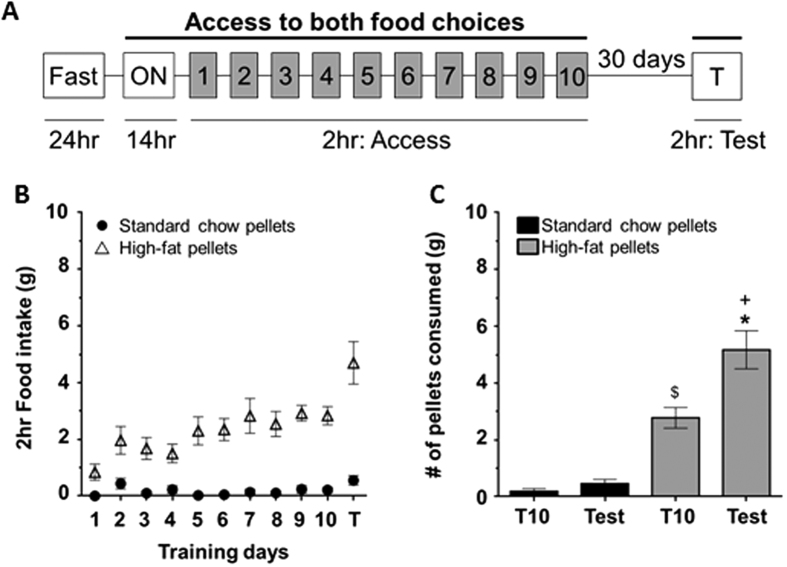
Rats showed an increased preference for high-fat pellets following a period of abstinence, as measured by a pellet preference test. (**A**) Experimental design for the pellet preference test (refer to methods). (**B)** Consumption of high-fat (HF, open triangles) and standard chow (SC, closed circles) pellets during training days and test. (**C)** Consumption of HF (gray) and SC (black) during training day 10 (T10) and test. By the end of training, rats ate significantly more HF than SC (^$^p < 0.05). Rats ate significantly more HF than SC on the test day after 30 days of abstinence (^+^p < 0.05) and consumed an even greater amount on test day than compared to T10 (*p < 0.05).

To determine whether rats prefer the HF pellets over the SC pellets, rats were given free access to both HF and SC pellets 2 hours daily for 10 days (training). Following training, rats were left undisturbed in the home cage for 30 days, during which they had access only to the standard LabDiet *ad libitum*. After the 30 days of abstinence, rats were again presented with the food choices (test) and consumption was measured. Rats showed a greater preference for the HF pellets compared to the SC pellets during the 10 training days, as measured by the relative increase in consumption of HF pellets (average daily consumption in grams: HF = 2.20 ± 0.20 (n = 8) vs. SC = 0.20 ± 0.04 (n = 8), F_(1, 7)_ = 34.45, p < 0.05, Fig. 1B). Furthermore, the preference for the HF pellets persisted over the abstinence period (consumption in grams: HF = 4.70 ± 0.70 vs SC = 0.60 ± 0.20; t (14) = 5.41, p < 0.05, Fig. 1C). Interestingly, the rats increased their consumption of HF pellets from the last training day to the test day (training day 10: 2.80 ± 0.30 compared to 4.70 ± 0.70; t (14) = 3.26, p < 0.05, Fig. 1C). We term this time-dependent increase in HF feeding “incubation of consumption”.

### Experiment 2: High-fat pellets induce conditioned place preference (CPP)

**Figure 2:**
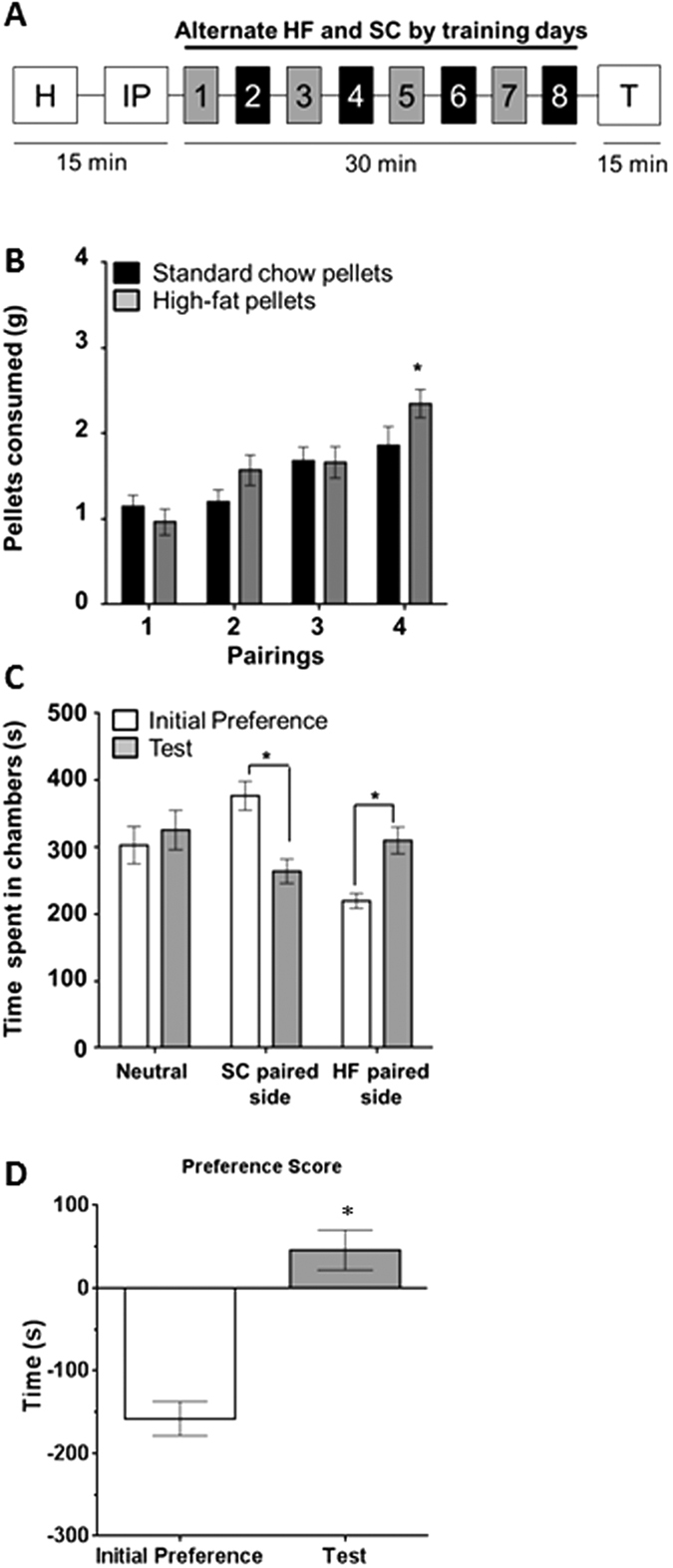
Rats demonstrated conditioned place preference for HF pellet rewards. (**A**) Experimental design of conditioned place preference (CPP). Grey boxes indicate training sessions with HF pellets and black boxes indicate training with SC. (**B**) Consumption of HF (gray) and SC (black) pellets during each pairing. Throughout training, rats significantly increased their consumption of the HF pellets compared to SC (*p < 0.05). (**C**) Time spent in each chamber during initial preference (white) and test (gray). The amount of time spent on the HF paired side during the test day was significantly higher than the initial preference day (*p < 0.05). (**D**) The CPP preference score was defined as the time spent (in seconds) on the HF-paired chamber during the CPP test minus the time spent on the HF-paired side during the IP day.

To further investigate the relative reward value of the 60% HF and SC pellets, we evaluated the ability of both pellet types to induce CPP. Rats were given free access to HF and SC pellets, paired in distinct chambers on alternating days for 8 days (Fig. 2A). By the end of the 8 days (4 pairings with each pellet type), rats consumed significantly more of the HF pellets (in grams: HF = 2.40 ± 0.20 compared to SC = 1.90 ± 0.20, n = 24, t (92) = 2.64, p < 0.05; Fig. 2B). We chose to utilize a biased design, such that the HF pellets were paired with the initially non-preferred chamber, which was determined in the initial preference (IP) test. Despite the fact that rats initially spent more time on the SC-paired side (time in seconds: IP: 377.10 ± 21.80, Test: 264.10 ± 18.10, t (46) = 3.98, p < 0.05; Fig. 2C), the HF pellets were sufficient to overcome this initial bias. After conditioning, rats spent significantly more time on the HF-paired side than they did during IP (time in seconds: IP: 219.74 ± 11.06, Test: 310.12 ± 20.04, t (46) = 3.95, p < 0.05; Fig. 2C). There was no difference in the amount of time spent in the neutral, unconditioned chamber between the IP and test day (IP: 303.14 ± 27.76, Test: 325.80 ± 29.44, t (46) = 0.56, Fig. 2C). These results indicate an increased preference for the HF paired chamber, as summarized by the preference score (F_(2, 69)_ = 13.59, p < 0.05; Fig. 2D).

### Experiment 3: Cues predictive of a food reward gain prominence over time

**Figure 3:**
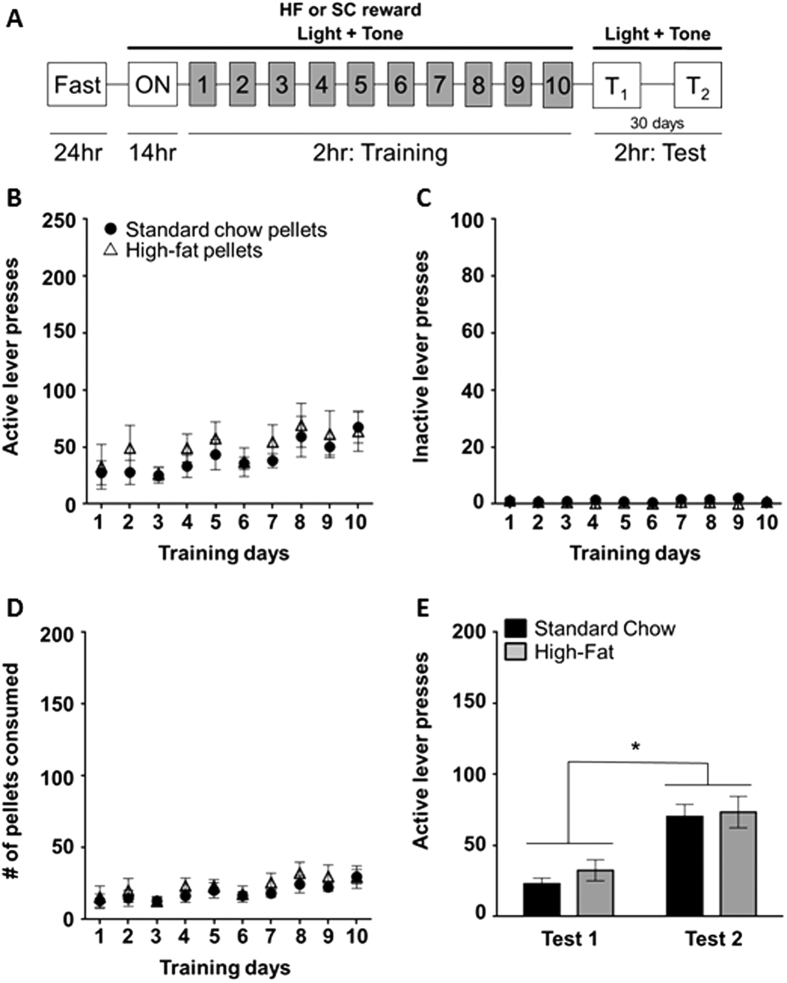
The associated cues maintained their prominence following a period of forced abstinence. **(A**) Experimental design for self-administration of food rewards (refer to methods). (**B–D**) Training responses for active lever presses (**B**), inactive lever presses (**C**), and reward pellets received (**D**) for either SC (closed circles) or HF (open triangles). (**E**) Summary of active lever presses during test days. Both SC and HF showed a significant increase in lever presses during Test 2 compared to Test 1 (*p < 0.05).

We next wanted to explore the potential for HF and SC pellets to induce incubation of craving, a phenomenon hypothesized to represent the motivation to reward seek. To do this, we utilized a self-administration paradigm in which rats were first trained to administer either HF or SC pellets and were subsequently left to lever press for the associated cues (light and tone) following short (1 day, Test 1) or prolonged (30 days, Test 2) abstinence. Given our observations in the pellet preference test and CPP assay, we anticipated that the SC pellets, being less preferred than the HF pellets, would not induce incubation of craving, providing us with a negative control. At day 10 of training, there was no significant difference in active lever pressing between HF and SC groups (Fig. 3B, Table 1). The same was observed with the number of rewards received (HF = 34.00 ± 6.00 (n = 9); SC = 32.00 ± 5.00 (n = 16); Fig. 3D). Additionally, there was no difference between groups for inactive lever pressing on day 10 of training, suggesting that both HF and SC groups were effectively able to discriminate between the levers and learn the behavioral task (SC = 0.70 ± 0.40; HF = 0.70 ± 0.20; Fig. 3C). During Test 1, in which the rats lever pressed for the associated cues only, there was no difference in the number of responses between the two groups (t (17) = 1.22; Fig. 3E, Table 1). To our surprise, there was a significant time-dependent increase in cue-seeking behavior in both HF and SC groups, as measured by the increase in active lever responding from Test 1 to Test 2 (F_(1, 17)_ = 59.93, p < 0.05; Fig. 3E, Table 1), indicating that both pellet types induce an incubation of craving effect. Moreover, there was no significant difference in responses between the two groups (t (17) = 4.52; Fig. 3E, Table 1) indicating the same incubation effect for both reward pellets.

### Experiment 4: Exclusive rearing with SC pellets blunts the development of the incubation of craving phenomenon

**Figure 4:**
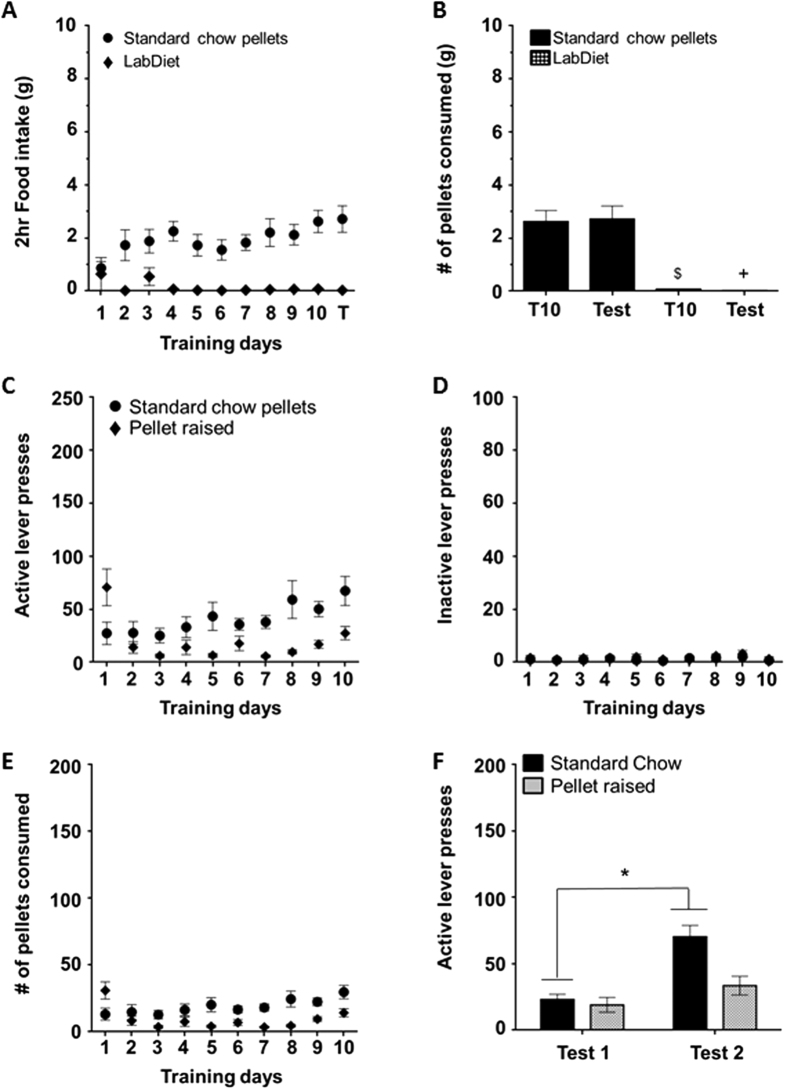
Rats demonstrated an increased preference for SC pellets compared to the LabDiet and when reared on SC pellets exclusively, rats failed to exhibit incubation of craving . (**A**) Consumption of SC pellets (closed circles) and LabDiet (closed diamonds) during the training days and test of a pellet preference test. Rats consumed significantly less of the LabDiet than the SC pellets during training (^$^p < 0.05). (**B**) Consumption of SC (black) and LabDiet (checkered) during training day 10 (T10) and test. Rats showed a significant preference for SC compared to LabDiet on the test day (^+^p < 0.05). (**C–E**) Self-administration training responses for SC pellets of pellet-raised (closed diamonds) and standard–raised (closed circles) rats for active lever presses (**C**), inactive lever presses (**D**), and SC reward pellets received (**E**). (**F**) Summary of active lever presses during test days. Pellet raised rats did not show a significant increase in lever presses between test dates, but SC rats did (*p<0.05).

Although the SC pellets closely matched the macronutrient profile of the home cage diet, given the results of our self-administration study, we hypothesized that the novelty and unique presentation of the pellets in the self-administration paradigm might have influenced their reward value. We tested this hypothesis by conducting a food preference test between the SC pellets and the home cage LabDiet. Rats demonstrated a significant preference for the SC pellets over the LabDiet during training (in grams: SC: 2.60 ± 0.40 vs. LabDiet: 0.07 ± 0.01, n = 8, F_(1, 7)_ = 40.03, p < 0.05, Fig. 4A), which persisted over the 30 days of abstinence (t (7) = 6.12, p < 0.05; Fig. 4A (T) and Fig. 4B). To further investigate this finding, we reared a cohort of rats on the SC pellets from weaning and subsequently placed them in the same self-administration paradigm described above to determine if incubation of SC would still be present. The pellet-raised rats pressed significantly less during training compared to the SC rats that were reared on the standard LabDiet (SC: n = 12, pellet raised: n = 7; pellet raised compared to SC: t (17) = 2.11, p < 0.05; Fig. 4C, Table 1). The same effect was observed with the number of rewards received (SC = 32.00 ± 5.00; pellet raised = 14.30 ± 3.00; Fig. 4E) and there was no difference between standard-raised and pellet-raised groups for inactive lever presses (SC = 0.70 ± 0.40; pellet raised = 1.20 ± 0.80; Fig. 4D). While there was no difference in lever pressing between groups during Test 1 (Fig. 4F; Table 1), SC-paired cues induced an incubation of craving effect, but only for animals in standard rearing conditions. Pellet-raised rats on the other hand failed to demonstrate incubation, indicated by the lack of increase in lever pressing from Test 1 to Test 2 (SC: t (17) = 7.081, p < 0.05; pellet raised: t (17) = 1.649, p = 0.22; Fig. 4F, Table 1). Our findings support the hypothesis that the SC pellets carried an unexpected reward value, likely attributable to their novel size and shape.

### Experiment 5: An altered motivational state does not impair the incubation of craving effect

**Figure 5:**
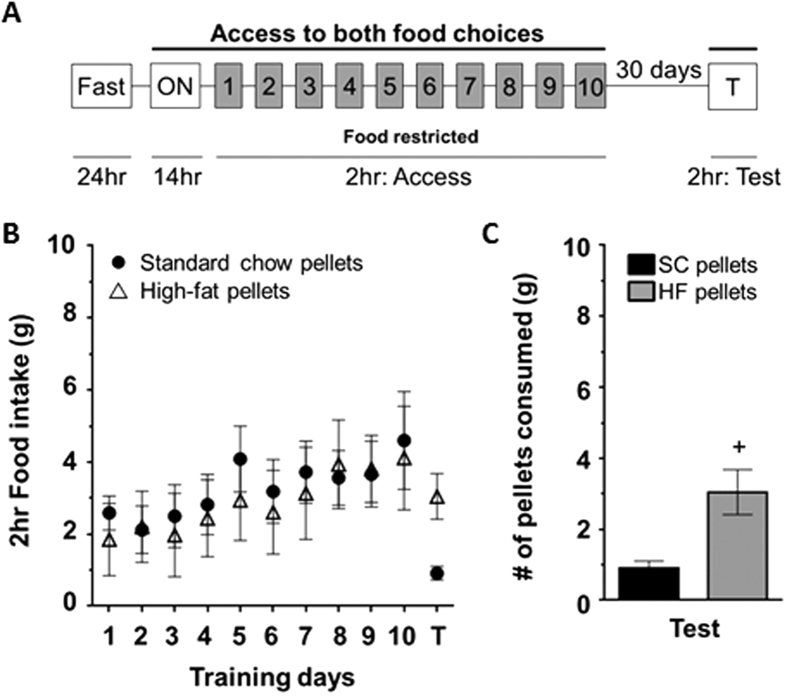
When trained in a food-restricted state, rats showed no preference for SC or HF pellets during initial training. However, following 30 days of *ad libitum* feeding, rats consumed more HF than SC. (**A**) Experimental design for the pellet preference testing (refer to methods). (**B**) Consumption of SC (closed circles) and HF (open triangles) pellets during training and test. (**C**) Consumption of SC (black) and HF (gray) during the test. Rats ate significantly more HF than SC on the test day after 30 days of abstinence (^+^p < 0.05).

We repeated the pellet preference test in a food restricted (FR) condition in order to look at the potential impact that an altered motivational state could have on the incubation effect. Rats trained in a FR state showed equal preference for the HF pellets and SC pellets (average daily consumption in grams: HF = 2.90 ± 0.30 (n = 8) vs. SC = 3.30 ± 0.30 (n = 8) respectively, F_(1, 7)_ = 0.06; Fig. 5B) throughout the 10 training days. Following 30 days of abstinence, during which time food was no longer restricted, rats consumed significantly more HF pellets (HF = 3.00 ± 0.60 vs. SC = 0.90 ± 0.20, t (14) = 3.25, p < 0.05; Fig. 5B (T) and Fig. 5C). The relative preference for HF compared to SC on test day (Fig. 5C) can be attributed to the fact that these rats were no longer food restricted during the abstinence period or during the test. As demonstrated in Experiment 1, rats in a satiated state will preferentially consume HF over SC. Interestingly, when trained in a FR state rats did not significantly increase their consumption of HF pellets from training day 10 to the test 30 days later, demonstrating that motivational state during training influences the incubation of consumption effect.

**Figure 6:**
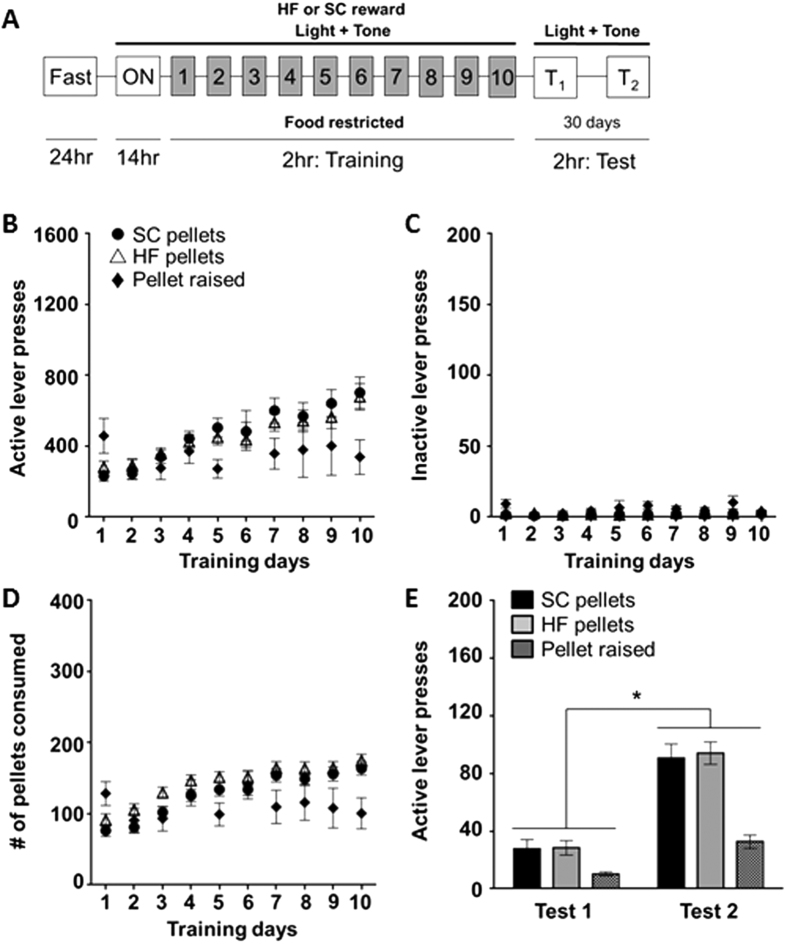
When rats are trained in a food-restricted state, cues predictive of reward gained prominence over time, regardless of reward type or rearing diet. (**A**) Experimental design for self-administration of food rewards (refer to methods). (**B–D**) Training responses of SC (closed circles), HF (open triangles), and pellet raised (closed diamonds) animals for active lever presses (**B**), inactive lever presses (**C**), and rewards received (**D**). (**E**) Summary of active lever presses during test days. All three groups showed a significant increase in active lever pressing from Test 1 compared to Test 2 (*p < 0.05).

We repeated the self-administration protocol as previously described (Fig. 3A) except that rats were kept in a FR state during training (Fig. 6A). Rats were subsequently tested in a satiated state 24 h after training (Test 1) for their responsiveness to the cues only. No difference in active lever pressing between SC and HF groups was observed, but both groups exhibited more active lever pressing compared to the pellet-raised rats (SC, n=20: t (26) = 1.71, p < 0.05; HF, n = 20: t (26) = 2.22, p < 0.05; Fig. 6E, Table 2). Furthermore, when they were tested 30 days later (Test 2), both HF and SC groups showed a significant increase in active lever pressing, or incubation of craving (F_(2, 90)_ = 3.129, p < 0.05; Fig. 6E, Table 2). Despite also demonstrating an incubation of craving, the pellet-raised group exhibited significantly less active lever pressing than the HF and SC groups during Test 2 (SC, n = 20: t (26) = 2.19, p < 0.05; HF, n = 20: t (26) = 3.17, p < 0.05; Fig. 4F, Table 2), further suggesting that when animals are reared on the standard LabDiet, distinct from the pellets in size and shape, cues predictive of SC and HF rewards become more salient than when animals are reared exclusively on SC pellets and the novelty of the SC pellets is absent. Importantly though, these results highlight the influence that motivational state during training has in eliciting an incubation of craving effect and support our hypothesis that the relative reward value perceived during training can initiate incubation independent of food palatability.

## Discussion

The purpose of this study was to determine whether exposure to HF-paired cues would elicit an incubation of craving effect as previously demonstrated for other rewarding stimuli^3^,^4^,^12^,^13^,^20^,^21^,^22^,^23^. “Craving” is often used to describe an intense desire people feel to seek out a stimulus of interest^24^ and tends to enhance the motivation to acquire that stimulus or its associated cues, particularly following a period of abstinence^25^,^26^,^27^. Similarly, in rodent self-administration models of addiction, it is suggested that an increase in approach behavior for drug-associated cues is reflective of an enhanced motivation to acquire that drug, and therefore models “craving”^4^. It has been repeatedly reported that animals will increase their behavioral responsiveness for cues previously paired with rewarding stimuli, including drugs of abuse but palatable food as well, and that this response will increase during a period of abstinence^3^,^4^,^5^,^10^,^28^,^29^. Importantly, if animals are given access to the reward again, they will consume it in greater amounts than they did prior to the period of abstinence^30^,^31^,^32^,^33^. This increase in consumption has been termed the “deprivation effect” and may contribute to relapse in drug addiction^2^,^34^,^35^,^36^,^37^. Given our results with high-fat and the fact that similar behaviors have been observed in response to other palatable foods, in both the rodent and human models^13^,^27^,^31^,^38^,^39^, it is reasonable to suggest that the induction of the incubation of craving phenomenon may, similarly to drugs of abuse, initiate a “relapse” to food, subsequently hindering the ability to successfully diet and thereby facilitating the development of obesity. Indeed, it has been suggested that a major hindrance in treating obesity is the presence of overwhelming cravings for palatable, fatty foods that override the ability to abstain from “unhealthy” food choices^38^,^39^. Several factors may contribute to this food “relapse” and cues, as demonstrated, are likely to play a significant role. The mechanisms that control the inability to ignore those cues are still under investigation. Recent work from our laboratory suggests that exposure to a HF diet attenuates dendritic spine density in the infralimbic prefrontal cortex (IL-PFC), attributable to a loss of thin type spines^40^. Given the role of this region in the inhibitory control of motivated behavior, this observation could contribute to challenges in dieting success in individuals who have had previous experience with excess fat. Furthermore, it has been demonstrated that selectively bred obesity-prone rats are more likely to over-consume “junk-food” high in sugar and fat content than obesity-resistant rats, attributed to striatal variance in dopamine^41^,^42^. Together, these studies indicate that exposure to dietary fat may induce neuronal adaptations, which could promote maladaptive feeding behaviors, particularly in individuals who are already overweight or who have had experience with excessively fatty food.

To our knowledge, this is the first study to report an incubation of craving effect for cues previously associated with dietary HF. In addition to HF, we examined whether cues associated with a SC pellet, manufactured to match the caloric profile of the home cage diet (LabDiet; 28.5% protein, 13.5% fat, 58.0% carbohydrate, Table 3), would induce an incubation of craving. We hypothesized that the rats exposed to the HF reward but not the SC reward would undergo this incubation of craving effect. However, both groups displayed equivalent magnitudes of incubation. Although this was unexpected, we believe this proved to be extremely insightful to the observed time-dependent increases that have been reported previously with lever pressing^4^,^12^,^35^. We speculate, based upon our data, that rodents will readily engage in behaviors that can be predictive of a reward outcome and that these behaviors become sensitized over time, which is influenced by the perceived reward value of the primary reinforcer (reward pellet). This is particularly important as the primary reinforcer may influence other aspects of reward processing (e.g., cortical control^43^ and alterations to dendritic spine morphology^40^), that could lead to maladaptive feeding behaviors.

In our first set of experiments, rats were given free choice between the control (SC) diet and the more “palatable” HF. There was no operant learning task associated with the training sessions. When kept in a food restricted state, rats did not show a preference between the control and the palatable food choice throughout the training sessions, likely due to a heightened interest in homeostatic food seeking. When they were given access to the food choices 30 days later in a satiated state, they demonstrated a preference for the more palatable food compared to the control. Conversely, when trained in a satiated state, rats showed a preference for the more palatable food during the training, in addition to the test day. This would imply that the motivation to consume the HF increased over time when the rats were satiated, which is consistent with literature showing that satiation level can directly impact reward value of food and may engage hedonic food selection^44^,^45^,^46^. The non-FR rats presented with HF made the distinction between the two pellet options from the start of training, choosing to consume the HF pellet over the SC pellet. Following 30 days of abstinence from the food pellets, rats consumed significantly more HF pellets than they did initially (Fig. 1). This time-dependent increase in consumption was not observed for SC. Previous work has shown that rats given *ad libitum* access to HF have the ability to self-regulate their caloric intake in the earlier stages of access, but eventually they will lose the self-regulation control and become obese^47^,^48^. We further assessed the reward value of HF using a CPP model and demonstrated a preference for HF compared to SC. Our data supports the findings that rats will prefer a more palatable food when satiated and increase their consumption for HF over time, which has important implications for the development of obesity. Mechanisms that may facilitate this hedonic consumption likely involve, as discussed, frontal dependent control of motivated behavior as well as dopamine variance in the nucleus accumbens.

In our operant experiments the reward pellet was paired with compound cues (i.e., a light and tone). This design mimicked previous experiments that evaluated liquid sucrose^11^,^12^,^13^. Our results showed a robust incubation of craving effect for HF and SC. Although we had the macronutrient profile of the SC pellet made to mimic the chow available in the home cage, when preference between the two choices (SC vs. LabDiet; Fig. 4) was assessed, rats preferred the SC pellets. This suggests that the 45 mg pellets are relatively more rewarding than LabDiet and may account for the incubation effect for SC pellets. It is hypothesized that the novel size and shape of the SC pellets (much smaller and spherical in comparison to the standard LabDiet bricks) contributed to the elevated reward value of the nutritionally similar diet and facilitated a craving-like behavior. Indeed, it has been demonstrated that the paired presentation of cues and a food pellet, even when similar in macronutrient composition to the home cage diet, can facilitate binge-like consummatory behavior^49^. Consequently, the rats’ previous food history and unique conditions of presentation may skew operant reward value independent of macronutrient profile. It should be noted though that novelty alone is not the primary contributor to the development of this phenomenon. In Experiment 5, we demonstrated that pellet-raised rats, who have had previous experience with the SC pellets, do exhibit incubation of craving when trained in a FR state (Fig. 6E), suggesting that the perceived value of a reward, rather than its novelty, is the driving force in its ability to induce an incubation effect.

We have shown that HF and SC have an incubation of craving effect, similar to sucrose. This is supported by the recent report that incubation of craving in response to sucrose self-administration is most likely due to the influence of the contextual cues paired with the reward^11^. This effect seems to be non-dependent upon the food reward. Our results support the hypothesis that contextual cues are a critical part of the incubation of craving phenomenon. The mechanisms that underlie this incubation, particularly related to high-fat, are presently under investigation but it has been demonstrated that the incubation of sucrose craving is associated with a decrease in AMPA/NMDA ratio in rats^23^. On the other hand, Brown *et al*.^50^, showed that obesity-prone rats, who self-administer more palatable food than obesity-resistant rats, also exhibit a greater α-amino-3-hydroxy-5-methyl-4-isoxazolepropionic acid (AMPA)/N-methyl-D-aspartate (NMDA) ratio, but these findings were not in the context of incubated craving^50^. Thus, the neuroadaptations that facilitate incubation of fat craving warrant further investigation.

Our results demonstrate that cues associated with food, independent of the macronutrient profile, which could influence palatability and preference, facilitate changes in rodent seeking behaviors over time reminiscent of an incubation of craving phenomenon. However, the palatability of the food significantly influences how much the animal actually consumes over time. Specifically, rodents with a history of HF food exposure show an increase in consumption after prolonged periods of abstinence (Fig. 1B). Taken together, we speculate that food cues gain importance over time, which engages elevated seeking behaviors and is independent of the macronutrient profile of the food. However, rats only consume more of the palatable food after abstinence when given a choice. Therefore, increased seeking in combination with an increase in consumption of HF over time may contribute to overeating and obesity. Finally, with the extensive use of the incubation of craving paradigm in evaluating reward-seeking behavior, these findings highlight the importance of proper controls in experiments that investigate approach behavior in rodents, whether it is related to drug administration or food reinforcement.

## Materials and Methods

### Subjects

All experiments were performed with male Sprague-Dawley rats, postnatal day 60 at the beginning of the experiment (body weight range: 300–350 g). Rats were housed individually in a temperature controlled room under a 12 h light/dark cycle. Rats had access to food and water at all times, except during food-restricted training and other training sessions described in detail below. All procedures were performed in accordance with the NIH *Guide for the Care and Use of Laboratory Animals* and with approval from the IACUC committee at the University of Wyoming.

### Pellet Preference Test

A pellet preference test was conducted to determine food preferences between the diets used in this study. Rats were fasted for 24 h prior to training in order to increase motivation for food seeking. They were placed in a Med Associates (St. Albans, VT) modular chamber (12 × 8.5 × 9 in) overnight for 14 h. Rats were provided with two feeding dishes placed in opposite corners of the chamber that contained either Bio-Serv (Flemington, NJ) grain based diet (#F0165; standard chow pellets, SC; 25.3% protein, 10.1% fat, 64.5% carbohydrate) and Bio-Serv purified diet 60% fat (#F07062; high-fat pellets, HF; 19.5% protein, 60.1% fat, 19.9% carbohydrate), or the home cage diet (Laboratory Rodent Diet #5001; St. Louis, MO; 28.5% protein, 13.5% fat, 58.0% carbohydrate). After the overnight session, rats were returned to their home cages with either 20 g (food restricted, FR) or 50 g (non-food restricted, Non-FR) of the standard LabDiet and remained in these varying satiation states for the remainder of training. They were placed in the same boxes each day for 2 h with access to the two dietary options. This was conducted in several iterations including HF vs. SC in both FR (Fig. 5) and non-FR states (Fig. 1), and SC vs. LabDiet in a non-FR state (Fig. 4). After 10 sessions, rats were placed in their home cages with *ad libitum* food access for 30 days. They were then returned to the box for 2 h with access to both diets as described above, and tested for their food preference, measured by consumption of food in grams.

### Conditioned Place Preference (CPP)

Conditioned place preference (CPP) was used to further validate food preference between the diets in this study and was conducted using a biased procedure^51^. Using Med Associates 3-tone boxes (white: 12 × 9 × 8.5″, grey: 5 × 9 × 8.5″, and black chambers: 12 × 9 × 8.5″); rats were placed in the middle grey chamber with free access to all three chambers for 15 min on the first day for habituation. The following day, the procedure was repeated to determine which side the rat naturally prefers, also termed the initial preference (IP). The conditioning sessions were conducted once per day for 8 consecutive days. On alternating days, rats were given access to the HF food pellets in the non-preferred side for 30 min or SC food pellets in the preferred side for 30 min. The post-conditioning session (Test) was conducted 24 h after the last training session in the same manner as the habituation and IP days (Fig. 2A). The CPP preference score was defined as the time spent (in seconds) on the HF-paired chamber during the CPP test minus the time spent on the HF-paired side during the IP day.

### Self-administration (SA)

To determine whether the various dietary rewards could induce an incubation of craving phenomenon a self-administration paradigm was utilized. Rats were fasted for 24 h to induce motivation for food seeking and were subsequently placed into an overnight training session (14 h) in Med Associates operant chambers (12 × 7 × 9.5″). Each box was equipped with two retractable levers located 6 cm above the grid floor and a house light, which remained illuminated throughout the duration of the session. A response on the active lever yielded the presentation of a food pellet reward (HF or SC), as well as a compound cue (illumination of a light located directly above the lever and a 5 s tone). Following a response on this lever, there was a 30 s time-out period; during which time lever presses were quantified but yielded no reward or cues. Presses on the inactive lever were also recorded, but did not have any programmed consequences. After this session, rats were returned to their home cages with either 50 g (Non-FR; Fig. 3A) or 20 g (FR; Fig. 6A) of the LabDiet and remained in these satiation states for the remainder of training. Training consisted of 2 h sessions each day for 10 days. Following the 10^th^ training session, all rats were given 50 g of food overnight before Test 1. In the first testing session, which followed exactly 24 hours after the last training session, conditions were identical as described above except that a response on the active lever yielded the presentation of the compound cue but not the reward. Following Test 1 rats were returned to their home cages for 29 days with *ad libitum* access to the LabDiet. Test 2 was performed on the 30^th^ day after training in the same manner as Test 1. Comparison of active lever pressing from Test 1 to Test 2 was used to determine the degree of incubation of food craving.

### Pellet-raised

In all of the above experiments, rats were given a standard rodent chow in the home cage environment. Although the SC pellets were manufactured to replicate the nutritional composition of standard home cage diet, our results indicated that the SC pellets may be perceived as more rewarding than the home cage food. To explore this hypothesis, a cohort of rats was given SC pellets in the home-cage environment from the day of weaning, and therefore never had exposure to the standard LabDiet. They subsequently followed the SA protocol described above. Half of the rats were FR (Fig. 6) during training and the other half were Non-FR (Fig. 4).

### Statistical analyses

The results are expressed as mean ± SEM. ANOVA was used with appropriate between- and within-subjects factors for the different experiments (see Results) and t-tests were used when necessary. Significant main effects and interactions (p < 0.05) in the factorial ANOVAs were further analyzed using Tukey’s *post hoc* test.

## Additional Information

**How to cite this article**: Darling, R. A. *et al*. Incubation of food craving is independent of macronutrient composition. *Sci. Rep.* **6**, 30900; doi: 10.1038/srep30900 (2016).

## Figures and Tables

**Table 1:** Active lever presses for non-food restricted training and test days.

**Day**	**SC Pellets**	**HF Pellets**	**Pellet Raised**
1	27.3 ± 10.5	32.7 ± 19.6	70.7 ± 17.2
2	27.7 ± 10.6	49.1 ± 20.0	14.0 ± 5.4
3	25.2 ± 6.8	26.4 ± 6.0	6.0 ± 1.8
4	33.1 ± 9.8	49.1 ± 12.3*	14.0 ± 6.8
5	43.3 ± 13.3	57.4 ± 14.7*	6.4 ± 1.8
6	35.7 ± 5.5	36.7 ± 12.4	17.7 ± 6.7
7	38.0 ± 6.0*	54.5 ± 14.8*	5.7 ± 1.1
8	59.0 ± 17.7*	69.1 ± 19.2*	9.7 ± 2.0
9	50.1 ± 7.1*	61.2 ± 20.5	16.8 ± 3.7
10	67.4 ± 13.7*	63.7 ± 17.4	27.4 ± 6.2
Test 1	23.1 ± 4.0	32.5 ± 7.3	19.1 ± 5.5
Test 2	70.4 ± 8.5^$^	73.5 ± 11.1^$^	31.5 ± 7.1

*p < 0.05: significance compared to the pellet raised group. ^$^p < 0.05: significance of Test 1 to Test 2.

**Table 2:** Active lever presses for food-restricted experiments.

**Day**	**SC Pellets**	**HF Pellets**	**Pellet Raised**
1	231.4 ± 40.7*	236.6 ± 32.3*	459.0 ± 98.2
2	227.7 ± 40.9	275.0 ± 29.0	270.9 ± 58.5
3	347.8 ± 54.1	344.2 ± 34.8	276.4 ± 64.7
4	467.2 ± 58.8	398.3 ± 52.8	371.0 ± 68.0
5	494.5 ± 63.4*	427.2 ± 60.9	272.8 ± 52.6
6	503.9 ± 73.4	416.8 ± 58.1	489.1 ± 112.8
7	679.3 ± 100.8	538.9 ± 70.1	358.1 ± 87.2
8	622.4 ± 102.7	526.9 ± 84.6	379.5 ± 155.2
9	688.8 ± 106.1	543.1 ± 92.4	401.6 ± 165.0
10	760.6 ± 107.3*	655.1 ± 94.7	339.0 ± 97.2
Test 1	27.8 v 6.4	28.4 ± 5.0	10.3 ± 1.3
Test 2	90.8 ± 9.6^$^	94.3 ± 7.8^$^	32.8 ± 4.6^$^

*p < 0.05: significance compared to the pellet raised group.

^$^p < 0.05: significance of Test 1 to Test 2.

**Table 3:** Nutritional composition of the home cage diet, standard chow pellets, and high-fat pellets.

Caloric Profile	Home Cage	Standard Chow	High-Fat
Carbohydrate	58.00	64.50	19.89
Fat	13.50	10.10	60.75
Protein	28.51	25.30	19.46
Fuel Value (kcal/g)	3.36	3.35	3.72
**Amino Acids (%)**			
Alanine	1.43	1.10	0.46
Arginine	1.41	1.08	0.64
Aspartic Acid	2.81	1.74	1.12
Cystine	0.31	0.28	0.35
Glutamic Acid	4.37	3.79	3.56
Glycine	1.21	0.85	0.43
Histidine	0.57	0.57	0.48
Isoleucine	1.14	1.02	0.96
Leucine	1.83	2.10	1.46
Lysine	1.41	1.10	1.30
Methionine	0.67	0.75	0.45
Phenylalanine	1.04	1.06	0.78
Proline	1.49	1.67	1.80
Serine	1.19	1.13	1.00
Threonine	0.91	0.84	0.77
Tryptophan	0.29	0.22	0.20
Tyrosine	0.71	0.81	1.00
Valine	1.17	1.15	1.14
**Vitamins (mg/kg)**			
Biotin	0.3	0.4	0.2
Choline	2,250.0	2,414.0	1,028.0
Folic Acid	7.1	11.4 (*as folate*)	2.0
Niacin	120.0	96.6	30.0
Pantothenic Acid	24.0	54.9	14.7
Pyridoxine	6.0	32.3	5.8
Riboflavin	4.5	31.9	6.0
Thiamin	16.0	31.4	6.0
Vitamin A (IU/Kg)	15,000.0	19,788.0	4,142.0
Vitamin B12 (IU/Kg)	50.0	50.0	25.0
Vitamin D3 (IU/Kg)	4,500.0	5,757.0	1,000.0
Vitamin E (IU/Kg)	130.0	136.0	77.4
**Fatty Acids (%)**			
Linoleic Acid	1.22	1.47	3.94
Linolenic Acid	0.10	0.14	0.80
Total Saturated	1.56	0.82	12.00
Total Monosaturated	1.60	1.06	8.05
